# Supplementary material for: Pilot-RCT Finds No Evidence for Modulation of Neuronal Networks of Auditory Hallucinations by Transcranial Direct Current Stimulation
Source: Brain Sci. 2022 Oct 12;12(10):1382. doi: 10.3390/brainsci12101382 (PMC9599056; doi:10.3390/brainsci12101382)
Supplement: Supplementary file 1 [file brainsci-12-01382-s001.zip › brainsci-1945179-supplementary.pdf]

Supplementary material

Methods

Table S1

Overview of the MRI sequences (in the order they were run) at baseline, post-treatment, and follow-up. Location I and location II are placeholders for the left DLPFC and TPC that were scanned in counterbalanced order.

**Sequence**	**Time (min:s)**
Structural T1 scan	07:07
MRS PRESS, location I	03:48
MRS MEGAPRESS, location I	10:12
MRS PRESS , location II	03:48
MRS MEGAPRESS, location II	10:12
Resting state fMRI	05:30
Dichotic listening/ fMRI	08:28
Arterial Spin Labelling*	04:14

Structural T1 scan, MRS, resting state fMRI: n=15; real/sham=9/6; Dichotic Listening/fMRI: n=11; real/sham= 6/5

**Not reported here.*

Results

Structural analysis

Exploratory t-tests were carried out for all 74 regions per hemisphere provided by the analysis comparing baseline and post-treatment for the whole group (Figure S1), as well as the real (FigureS 2) and sham group (Figure S3) separately, for three anatomical measures: surface area, gray matter volume, and average cortical thickness. The presented *p*-values in Figure S1, S2, and S3 are not corrected for multiple testing, and all are above 0.001 (the most significant being $10^{-2.8}$= 0.0016). So, none would reach significance if, for instance, Bonferroni correction for 444 variables was done.

Figure S1

Overview of t-tests comparing the whole group between baseline and treatment surface area, gray matter volume, and average cortical thickness for all 74 brain regions per hemisphere.

All Subjects (L/R), -log10(pvalue)

	prepost.lh.SurfArea	prepost.lh.GrayVol	prepost.lh.ThickAvg	prepost.rh.SurfArea	prepost.rh.GrayVol	prepost.rh.ThickAvg	
G_and_S_frontomargin	0.2	0.1	0.0	0.4	0.2	0.2	G_and_S_frontomargin
G_and_S_occipital_inf	0.9	1.2	0.5	0.4	0.3	0.2	G_and_S_occipital_inf
G_and_S_paracentral	0.7	0.8	0.2	0.1	0.7	0.6	G_and_S_paracentral
G_and_S_subcentral	0.1	1.1	0.2	1.2	1.1	0.2	G_and_S_subcentral
_and_S_transv_frontopol	0.2	0.0	0.4	1.0	0.2	0.8	G_and_S_transv_frontop
G_and_S_cingul-Ant	0.1	0.1	0.1	0.3	0.3	0.0	G_and_S_cingul-Ant
G_and_S_cingul-Mid-Ant	1.1	1.5	0.5	1.1	1.3	0.4	G_and_S_cingul-Mid-Ant
_and_S_cingul-Mid-Post	0.6	0.4	0.1	0.2	0.3	0.1	G_and_S_cingul-Mid-Pos
G_cingul-Post-dorsal	0.0	0.0	0.1	0.6	0.2	0.6	G_cingul-Post-dorsal
G_cingul-Post-ventral	0.6	0.5	0.1	0.3	0.3	0.2	G_cingul-Post-ventral
G_cuneus	0.7	0.0	0.5	0.0	0.1	0.2	G_cuneus
G_front_inf-Opercular	0.6	0.5	0.0	0.2	0.2	0.8	G_front_inf-Opercular
G_front_inf-Orbital	0.2	0.3	0.2	0.7	0.3	0.0	G_front_inf-Orbital
G_front_inf-Triangul	0.5	0.7	0.4	0.4	0.5	0.2	G_front_inf-Triangul
G_front_middle	0.2	0.7	0.2	0.5	0.3	0.3	G_front_middle
G_front_sup	1.2	0.6	0.0	0.9	0.5	0.5	G_front_sup
_Ins_lg_and_S_cent_ins	0.3	0.9	0.4	0.1	0.3	0.3	G_Ins_lg_and_S_cent_in
G_insular_short	1.3	0.1	0.4	0.1	0.0	0.2	G_insular_short
G_occipital_middle	0.2	0.1	0.3	0.6	0.6	0.0	G_occipital_middle
G_occipital_sup	0.1	0.2	0.1	0.3	0.1	0.3	G_occipital_sup
G_oc-temp_lat-fusifor	0.9	0.4	0.7	1.3	0.1	1.7	G_oc-temp_lat-fusifor
G_oc-temp_med-Lingual	0.6	0.0	0.3	0.7	0.3	0.8	G_oc-temp_med-Lingual
G_oc-temp_med-Parahip	0.4	0.0	0.3	0.1	0.2	0.0	G_oc-temp_med-Parahip
G_orbital	0.6	0.3	0.0	0.1	0.1	0.1	G_orbital
G_pariet_inf-Angular	0.7	0.3	0.2	0.6	0.1	0.4	G_pariet_inf-Angular
G_pariet_inf-Supramar	1.0	0.2	0.2	0.0	0.3	0.5	G_pariet_inf-Supramar
G_parietal_sup	0.1	0.2	0.6	0.0	0.4	0.6	G_parietal_sup
G_postcentral	0.3	0.6	0.2	0.5	0.2	0.1	G_postcentral
G_precentral	0.0	0.0	0.1	0.2	0.7	0.6	G_precentral
G_precuneus	0.7	0.5	0.2	0.4	0.0	0.2	G_precuneus
G_rectus	0.0	0.1	0.1	0.8	1.1	0.2	G_rectus
G_subcallosal	0.1	0.1	0.7	0.2	0.4	0.1	G_subcallosal
G_temp_sup-G_T_transv	0.1	0.0	0.1	0.1	0.1	0.2	G_temp_sup-G_T_transv
G_temp_sup-Lateral	0.8	1.3	1.0	0.1	0.5	0.2	G_temp_sup-Lateral
G_temp_sup-Plan_polar	0.2	0.2	0.8	0.4	0.6	0.3	G_temp_sup-Plan_polar
G_temp_sup-Plan_tempo	0.0	0.0	0.1	1.0	1.2	0.6	G_temp_sup-Plan_tempo
G_temporal_inf	0.3	0.1	0.7	0.3	1.1	0.3	G_temporal_inf
G_temporal_middle	1.0	0.5	0.6	0.0	0.3	0.2	G_temporal_middle
Lat_Fis-ant-Horizont	1.2	1.2	0.8	0.4	0.0	0.4	Lat_Fis-ant-Horizont
Lat_Fis-ant-Vertical	0.2	0.3	0.5	0.1	0.1	0.1	Lat_Fis-ant-Vertical
Lat_Fis-post	0.2	0.1	0.1	1.2	0.6	0.0	Lat_Fis-post
Pole_occipital	0.3	0.7	0.2	0.2	0.1	0.2	Pole_occipital
Pole_temporal	0.5	0.1	0.3	0.2	0.3	0.6	Pole_temporal
S_calcarine	0.2	0.1	0.4	0.1	0.2	0.5	S_calcarine
S_central	0.8	0.3	0.2	0.9	1.1	0.5	S_central
S_cingul-Marginalis	0.2	0.1	0.5	0.3	0.4	0.2	S_cingul-Marginalis
S_circular_insula_ant	0.0	0.2	1.4	0.1	0.1	0.2	S_circular_insula_ant
S_circular_insula_inf	0.2	0.2	0.1	0.3	0.2	0.5	S_circular_insula_inf
S_circular_insula_sup	0.5	0.5	0.0	0.3	0.1	2.1	S_circular_insula_sup
S_collat_transv_ant	0.0	0.0	0.0	0.9	0.7	0.3	S_collat_transv_ant
S_collat_transv_post	0.0	0.0	0.3	0.1	0.2	0.2	S_collat_transv_post
S_front_inf	0.1	0.1	0.6	0.2	0.3	0.2	S_front_inf
S_front_middle	1.4	1.9	1.5	0.2	0.2	0.3	S_front_middle
S_front_sup	0.1	0.1	0.6	0.4	0.4	0.2	S_front_sup
S_interm_prim-Jensen	0.8	0.6	0.0	0.2	0.4	0.1	S_interm_prim-Jensen
_intrapariet_and_P_trans	0.6	0.6	0.2	0.1	0.1	0.0	S_intrapariet_and_P_tran
_oc_middle_and_Lunatus	0.1	0.3	0.1	0.2	0.1	0.2	S_oc_middle_and_Lunatu
_oc_sup_and_transversal	0.8	1.0	0.1	0.5	0.4	0.3	S_oc_sup_and_transvers
S_occipital_ant	0.2	0.5	0.5	-0.0	0.2	0.2	S_occipital_ant
S_oc-temp_lat	0.4	0.5	0.1	1.2	1.4	0.0	S_oc-temp_lat
-temp_med_and_Lingual	0.6	0.4	0.2	0.2	0.3	0.2	S_oc-temp_med_and_Lin
S_orbital_lateral	0.5	0.1	0.3	0.1	0.0	0.1	S_orbital_lateral
S_orbital_med-olfact	0.6	0.1	0.3	0.1	0.1	0.3	S_orbital_med-olfact
S_orbital-H_Shaped	0.7	0.7	0.3	0.3	0.2	0.1	S_orbital-H_Shaped
S_parieto_occipital	0.0	0.1	0.1	0.2	0.2	0.3	S_parieto_occipital
S_pericallosal	0.0	0.1	0.2	0.1	0.5	0.5	S_pericallosal
S_postcentral	0.3	0.5	0.3	0.8	0.8	0.3	S_postcentral
S_precentral-inf-part	0.7	1.1	0.2	0.4	0.4	0.1	S_precentral-inf-part
S_precentral-sup-part	0.7	0.2	1.0	0.2	0.1	0.4	S_precentral-sup-part
S_suborbital	0.5	0.3	0.5	0.1	0.1	0.2	S_suborbital
S_subparietal	0.6	0.7	0.0	0.0	0.0	0.0	S_subparietal
S_temporal_inf	0.5	0.0	0.9	0.3	0.1	0.9	S_temporal_inf
S_temporal_sup	0.1	0.0	0.2	0.1	0.1	0.0	S_temporal_sup
S_temporal_transverse	0.4	0.5	0.7	0.5	0.6	0.5	S_temporal_transverse

Note. SurfArea = surface area; GrayVol = gray matter volume; ThickAvg = average cortical thickness; lh = left hemisphere; rh = right hemisphere. *p*-values are expressed as the negative exponents of ten. For example, 0.2 translates into $p = 10^{-0.2} = 0.631$). This corresponds with approximately 1.3 for p=0.05, 2.0 for p=0.01, and 3.0 for p=0.001.

Figure S2

T-tests for all the 74 regions per hemisphere in the structural analysis in the real tDCS group (Stim) between baseline and post-treatment are given for surface area, gray matter volume, and average cortical thickness.

Stim (L/R), -log10(pvalue)

	prepost.lh.SurfArea	prepost.lh.GrayVol	prepost.lh.ThickAvg	prepost.rh.SurfArea	prepost.rh.GrayVol	prepost.rh.ThickAvg	
G_and_S_frontomargin	0.5	0.0	0.0	1.2	0.6	0.2	G_and_S_frontomargin
G_and_S_occipital_inf	1.2	1.1	0.2	0.5	0.2	0.8	G_and_S_occipital_inf
G_and_S_paracentral	0.7	0.6	0.2	0.2	0.3	0.4	G_and_S_paracentral
G_and_S_subcentral	0.0	0.4	0.1	2.6	2.3	0.3	G_and_S_subcentral
_and_S_transv_frontopol	0.2	0.1	1.3	1.3	0.8	0.7	G_and_S_transv_frontop
G_and_S_cingul-Ant	0.6	0.2	0.0	0.7	0.8	0.1	G_and_S_cingul-Ant
G_and_S_cingul-Mid-Ant	0.3	0.8	0.3	0.6	0.9	0.1	G_and_S_cingul-Mid-Ant
_and_S_cingul-Mid-Post	0.6	0.5	0.3	0.2	0.3	0.2	G_and_S_cingul-Mid-Pos
G_cingul-Post-dorsal	0.4	0.1	0.3	0.2	0.3	0.3	G_cingul-Post-dorsal
G_cingul-Post-ventral	0.3	0.3	0.2	0.2	0.2	0.1	G_cingul-Post-ventral
G_cuneus	0.1	0.2	0.1	0.4	0.1	0.3	G_cuneus
G_front_inf-Opercular	0.2	0.5	0.1	0.0	0.7	0.6	G_front_inf-Opercular
G_front_inf-Orbital	0.9	0.7	0.5	0.2	0.0	0.1	G_front_inf-Orbital
G_front_inf-Triangul	2.0	0.9	0.0	0.0	0.1	0.0	G_front_inf-Triangul
G_front_middle	0.1	0.8	0.2	0.5	0.1	1.6	G_front_middle
G_front_sup	0.6	0.0	0.1	0.9	0.8	0.3	G_front_sup
_Ins_lg_and_S_cent_ins	0.7	0.9	0.0	0.4	0.4	0.0	G_Ins_lg_and_S_cent_in
G_insular_short	0.6	0.0	0.5	0.2	0.3	0.4	G_insular_short
G_occipital_middle	0.1	0.1	0.5	0.5	0.4	0.1	G_occipital_middle
G_occipital_sup	0.2	0.6	0.4	0.5	0.7	0.1	G_occipital_sup
G_oc-temp_lat-fusifor	0.1	0.0	0.1	1.1	0.1	2.3	G_oc-temp_lat-fusifor
G_oc-temp_med-Lingual	0.1	0.2	0.4	0.7	0.2	0.7	G_oc-temp_med-Lingual
G_oc-temp_med-Parahip	2.0	0.2	0.6	0.3	0.7	0.0	G_oc-temp_med-Parahip
G_orbital	0.1	0.5	0.4	0.1	0.0	0.2	G_orbital
G_pariet_inf-Angular	0.9	0.2	0.2	0.1	0.1	0.5	G_pariet_inf-Angular
G_pariet_inf-Supramar	0.4	0.3	0.2	0.3	0.7	0.6	G_pariet_inf-Supramar
G_parietal_sup	0.3	0.1	0.8	0.2	0.7	0.8	G_parietal_sup
G_postcentral	0.2	0.1	0.2	0.8	0.0	0.4	G_postcentral
G_precentral	0.1	0.2	0.1	0.2	1.8	0.6	G_precentral
G_precuneus	0.5	0.6	0.0	0.2	0.1	0.2	G_precuneus
G_rectus	1.7	0.4	0.0	0.2	0.7	0.8	G_rectus
G_subcallosal	0.3	0.8	0.6	0.1	0.0	0.2	G_subcallosal
G_temp_sup-G_T_transv	0.3	0.5	0.0	0.0	0.1	0.1	G_temp_sup-G_T_transv
G_temp_sup-Lateral	0.3	0.5	0.7	0.1	0.5	0.2	G_temp_sup-Lateral
G_temp_sup-Plan_polar	0.1	0.1	0.1	0.7	1.2	0.4	G_temp_sup-Plan_polar
G_temp_sup-Plan_tempo	0.2	0.2	0.3	0.2	0.3	0.6	G_temp_sup-Plan_tempo
G_temporal_inf	0.0	0.0	0.1	0.0	0.3	0.1	G_temporal_inf
G_temporal_middle	0.3	0.5	0.3	0.3	0.1	0.2	G_temporal_middle
Lat_Fis-ant-Horizont	0.6	0.7	0.9	0.8	1.0	0.2	Lat_Fis-ant-Horizont
Lat_Fis-ant-Vertical	0.2	0.7	0.7	0.1	0.4	0.2	Lat_Fis-ant-Vertical
Lat_Fis-post	0.7	0.0	0.5	1.0	0.6	0.1	Lat_Fis-post
Pole_occipital	0.7	0.8	0.2	0.8	0.2	0.0	Pole_occipital
Pole_temporal	0.2	0.4	0.1	0.1	0.5	0.5	Pole_temporal
S_calcarine	0.1	0.1	0.1	0.7	0.3	0.3	S_calcarine
S_central	0.1	0.1	0.4	0.6	0.5	0.2	S_central
S_cingul-Marginalis	0.2	0.1	0.5	0.3	0.4	0.1	S_cingul-Marginalis
S_circular_insula_ant	0.0	0.3	1.6	0.2	0.4	0.5	S_circular_insula_ant
S_circular_insula_inf	0.0	0.2	0.2	0.0	0.1	0.3	S_circular_insula_inf
S_circular_insula_sup	0.3	0.3	0.2	0.0	0.2	1.1	S_circular_insula_sup
S_collat_transv_ant	0.2	0.1	0.1	0.4	0.0	0.5	S_collat_transv_ant
S_collat_transv_post	0.0	0.1	0.0	0.3	0.2	0.4	S_collat_transv_post
S_front_inf	0.0	0.1	0.0	1.2	0.6	0.4	S_front_inf
S_front_middle	0.6	1.0	1.3	0.1	0.1	1.0	S_front_middle
S_front_sup	0.1	0.0	1.1	0.4	0.5	0.3	S_front_sup
S_interm_prim-Jensen	0.1	0.1	0.2	0.3	0.1	0.3	S_interm_prim-Jensen
_intrapariet_and_P_trans	0.3	0.4	0.6	0.0	0.4	0.7	S_intrapariet_and_P_tran
_oc_middle_and_Lunatus	0.1	0.5	0.3	0.2	0.4	0.5	S_oc_middle_and_Lunatu
_oc_sup_and_transversal	0.4	0.7	0.5	0.8	1.2	0.3	S_oc_sup_and_transvers
S_occipital_ant	0.0	0.2	0.0	0.0	0.3	0.6	S_occipital_ant
S_oc-temp_lat	0.4	0.6	0.7	0.4	0.5	0.4	S_oc-temp_lat
-temp_med_and_Lingual	0.4	0.4	0.1	0.8	1.4	0.4	S_oc-temp_med_and_Lin
S_orbital_lateral	0.1	0.0	0.0	0.0	0.1	1.0	S_orbital_lateral
S_orbital_med-olfact	0.1	0.2	0.3	0.1	0.0	0.1	S_orbital_med-olfact
S_orbital-H_Shaped	0.5	0.2	0.0	0.8	1.1	0.1	S_orbital-H_Shaped
S_parieto_occipital	0.0	0.0	0.2	0.2	0.1	0.0	S_parieto_occipital
S_pericallosal	0.2	0.3	0.4	0.4	0.7	0.3	S_pericallosal
S_postcentral	0.1	0.4	1.0	0.1	0.0	0.0	S_postcentral
S_precentral-inf-part	0.1	0.8	1.0	0.7	0.8	0.1	S_precentral-inf-part
S_precentral-sup-part	0.3	0.0	1.0	0.3	0.2	1.8	S_precentral-sup-part
S_suborbital	0.8	0.6	0.3	0.1	0.2	1.0	S_suborbital
S_subparietal	0.1	0.3	0.2	0.6	0.7	0.0	S_subparietal
S_temporal_inf	0.4	0.0	0.5	0.2	0.6	1.2	S_temporal_inf
S_temporal_sup	0.1	0.2	0.2	0.1	0.1	0.0	S_temporal_sup
S_temporal_transverse	0.3	0.2	0.1	0.1	0.1	0.2	S_temporal_transverse

Note. SurfArea = surface area; GrayVol = gray matter volume; ThickAvg = average cortical thickness; lh = left hemisphere; rh = right hemisphere. *p*-values are expressed as the negative exponents of ten.

Figure S3

T-tests for all the 74 regions per hemisphere in the structural analysis in the sham tDCS group (Sham) between baseline and post-treatment are given for surface area, gray matter volume, and average cortical thickness.

Sham (L/R), -log10(pvalue)

	prepost.lh.SurfArea	prepost.lh.GrayVol	prepost.lh.ThickAvg	prepost.rh.SurfArea	prepost.rh.GrayVol	prepost.rh.ThickAvg	
G_and_S_frontomargin	1.1	0.2	0.0	0.1	0.2	0.0	G_and_S_frontomargin
G_and_S_occipital_inf	0.0	0.3	0.3	0.0	0.2	0.1	G_and_S_occipital_inf
G_and_S_paracentral	0.2	0.4	0.1	0.1	0.7	0.4	G_and_S_paracentral
G_and_S_subcentral	0.2	0.8	0.2	0.0	0.0	0.2	G_and_S_subcentral
_and_S_transv_frontopol	0.1	0.2	0.5	0.2	0.3	0.3	G_and_S_transv_frontop
G_and_S_cingul-Ant	0.4	0.4	0.1	0.4	0.4	0.0	G_and_S_cingul-Ant
G_and_S_cingul-Mid-Ant	1.2	0.9	0.3	0.7	0.7	0.8	G_and_S_cingul-Mid-Ant
_and_S_cingul-Mid-Post	0.1	0.0	0.1	0.1	0.1	0.1	G_and_S_cingul-Mid-Pos
G_cingul-Post-dorsal	0.7	0.1	0.8	0.5	0.1	0.5	G_cingul-Post-dorsal
G_cingul-Post-ventral	0.4	0.5	0.0	0.1	0.2	0.4	G_cingul-Post-ventral
G_cuneus	0.9	0.1	0.5	1.1	0.0	0.5	G_cuneus
G_front_inf-Opercular	0.4	0.1	0.3	0.4	0.2	0.3	G_front_inf-Opercular
G_front_inf-Orbital	1.5	0.9	0.3	1.0	0.6	0.1	G_front_inf-Orbital
G_front_inf-Triangul	0.0	0.2	0.9	1.0	1.0	0.2	G_front_inf-Triangul
G_front_middle	0.1	0.3	0.0	0.1	0.7	0.9	G_front_middle
G_front_sup	0.8	1.2	0.4	0.0	0.1	0.3	G_front_sup
_Ins_lg_and_S_cent_ins	0.6	0.2	0.5	0.3	0.0	0.5	G_Ins_lg_and_S_cent_in
G_insular_short	1.8	0.2	0.0	0.0	0.3	0.4	G_insular_short
G_occipital_middle	0.6	0.2	0.6	0.3	0.3	0.0	G_occipital_middle
G_occipital_sup	0.5	0.3	0.3	0.6	0.4	0.3	G_occipital_sup
G_oc-temp_lat-fusifor	1.1	0.6	0.8	0.5	0.0	0.4	G_oc-temp_lat-fusifor
G_oc-temp_med-Lingual	0.8	0.5	1.8	0.1	0.3	0.2	G_oc-temp_med-Lingual
G_oc-temp_med-Parahip	0.3	0.2	0.0	1.1	1.0	0.0	G_oc-temp_med-Parahip
G_orbital	0.7	0.1	0.8	0.0	0.1	0.1	G_orbital
G_pariet_inf-Angular	0.0	0.2	0.4	1.1	0.4	0.1	G_pariet_inf-Angular
G_pariet_inf-Supramar	0.7	0.0	0.5	0.2	0.4	0.1	G_pariet_inf-Supramar
G_parietal_sup	0.3	0.1	0.1	0.2	0.3	0.1	G_parietal_sup
G_postcentral	0.2	0.9	0.9	0.1	0.2	0.1	G_postcentral
G_precentral	0.1	0.3	0.6	0.0	0.0	0.1	G_precentral
G_precuneus	0.3	0.0	0.2	0.3	0.1	0.1	G_precuneus
G_rectus	0.5	0.5	0.1	1.6	0.6	0.4	G_rectus
G_subcallosal	0.4	0.6	0.2	0.2	0.6	0.6	G_subcallosal
G_temp_sup-G_T_transv	0.2	0.3	0.1	0.1	0.0	0.1	G_temp_sup-G_T_transv
G_temp_sup-Lateral	0.6	0.9	0.5	0.1	0.2	0.0	G_temp_sup-Lateral
G_temp_sup-Plan_polar	0.3	0.4	1.0	0.2	0.2	0.1	G_temp_sup-Plan_polar
G_temp_sup-Plan_tempo	0.2	0.2	0.2	2.5	1.9	0.1	G_temp_sup-Plan_tempo
G_temporal_inf	0.8	0.2	1.2	0.5	0.9	0.4	G_temporal_inf
G_temporal_middle	1.1	0.1	0.9	0.3	0.4	0.3	G_temporal_middle
Lat_Fis-ant-Horizont	0.9	0.7	0.3	0.3	0.9	0.9	Lat_Fis-ant-Horizont
Lat_Fis-ant-Vertical	0.0	0.0	0.1	0.0	0.1	0.2	Lat_Fis-ant-Vertical
Lat_Fis-post	0.2	0.1	0.4	0.4	0.1	0.2	Lat_Fis-post
Pole_occipital	0.1	0.0	0.1	0.6	0.0	0.2	Pole_occipital
Pole_temporal	0.3	0.1	0.4	0.4	0.0	0.3	Pole_temporal
S_calcarine	0.2	0.1	0.5	0.3	0.5	0.3	S_calcarine
S_central	2.8	0.6	0.2	0.5	1.0	0.5	S_central
S_cingul-Marginalis	0.1	0.0	0.1	0.1	0.1	0.1	S_cingul-Marginalis
S_circular_insula_ant	0.0	0.0	0.0	0.4	0.4	0.4	S_circular_insula_ant
S_circular_insula_inf	0.5	0.1	0.1	0.4	0.3	0.3	S_circular_insula_inf
S_circular_insula_sup	0.4	0.4	0.3	0.7	0.6	1.5	S_circular_insula_sup
S_collat_transv_ant	0.2	0.1	0.1	0.7	1.3	0.3	S_collat_transv_ant
S_collat_transv_post	0.0	0.1	0.3	0.1	0.8	0.6	S_collat_transv_post
S_front_inf	0.2	0.1	1.5	0.3	0.1	0.7	S_front_inf
S_front_middle	1.1	1.2	0.4	0.1	0.4	0.4	S_front_middle
S_front_sup	0.0	0.2	0.0	0.0	0.1	0.4	S_front_sup
S_interm_prim-Jensen	1.0	0.7	0.2	0.6	0.4	0.1	S_interm_prim-Jensen
_intraparietal_and_P_trans	0.5	0.4	0.1	0.5	0.5	0.4	S_intraparietal_and_P_tran
_oc_middle_and_Lunatus	0.1	0.1	0.3	1.0	0.8	0.3	S_oc_middle_and_Lunatu
_oc_sup_and_transversal	0.5	0.5	0.3	0.0	0.2	0.5	S_oc_sup_and_transvers
S_occipital_ant	0.2	0.4	0.8	0.0	0.0	0.1	S_occipital_ant
S_oc-temp_lat	0.1	0.1	1.1	1.1	1.6	0.8	S_oc-temp_lat
-temp_med_and_Lingual	0.5	0.0	0.4	0.5	0.4	0.1	S_oc-temp_med_and_Lin
S_orbital_lateral	0.7	0.2	0.4	0.2	0.1	0.8	S_orbital_lateral
S_orbital_med-olfact	0.7	0.0	0.2	0.3	0.2	0.4	S_orbital_med-olfact
S_orbital-H_Shaped	0.5	0.6	0.6	0.6	0.9	0.2	S_orbital-H_Shaped
S_parieto_occipital	0.0	0.2	0.1	0.5	0.1	0.4	S_parieto_occipital
S_pericallosal	0.4	0.3	0.2	0.5	0.1	0.6	S_pericallosal
S_postcentral	0.9	0.1	0.7	1.0	1.0	0.8	S_postcentral
S_precentral-inf-part	1.0	0.4	1.0	0.4	0.4	0.2	S_precentral-inf-part
S_precentral-sup-part	0.6	0.6	0.2	0.1	0.3	0.4	S_precentral-sup-part
S_suborbital	0.0	0.1	0.4	0.4	0.6	0.4	S_suborbital
S_subparietal	0.8	0.9	0.1	0.1	0.2	0.0	S_subparietal
S_temporal_inf	0.2	0.0	0.5	0.4	0.2	0.1	S_temporal_inf
S_temporal_sup	0.0	0.3	0.6	0.2	0.2	0.0	S_temporal_sup
S_temporal_transverse	0.9	1.2	1.0	0.8	0.5	1.1	S_temporal_transverse

Note. SurfArea = surface area; GrayVol = gray matter volume; ThickAvg = average cortical thickness; lh = left hemisphere; rh = right hemisphere. *p*-values are expressed as the negative exponents of ten.

MR spectroscopy

The means for Glx and GABA across all time points for both real and sham tDCS groups are presented in Table S2. Figure S2 depicts the voxel placement (panel A), a typical MR spectroscopy spectrum as provided by LCModel (panel B), and mean Glx and GABA levels for the DLPFC and TPC in real and sham participants across baseline, post-treatment, and follow-up (panels C to F).

None of the other metabolites or parameters (choline, creatine, myo-inositol and NAA) showed a significant main effect or interaction involving the factor Stimulation. The SNR did not differ between groups (all $Fs_{(1,18)} \leq 2.00$, $ps \geq .182$, $\eta_p^2 s \leq .143$).

Table S2

Descriptive means for Glx and GABA by real and sham group (values in institutional units).

**Test**	**Baseline**		**Post-treatment**		**Follow-up**	
	**Real**	**Sham**	**Real**	**Sham**	**Real**	**Sham**
Glx DLPFC	13.0	13.6	12.4	14.1	12.2	13.6
Glx TPC	12.6	12.4	12.6	12.7	12.7	14.0
GABA DLPFC	2.5	2.3	2.5	2.6	2.4	2.2
GABA TPC	2.3	2.5	2.4	2.3	2.4	2.5

Glx – glutamine + glutamate, GABA - γ-aminobutyric acid.

Figure S4

MR spectroscopy setup and results.

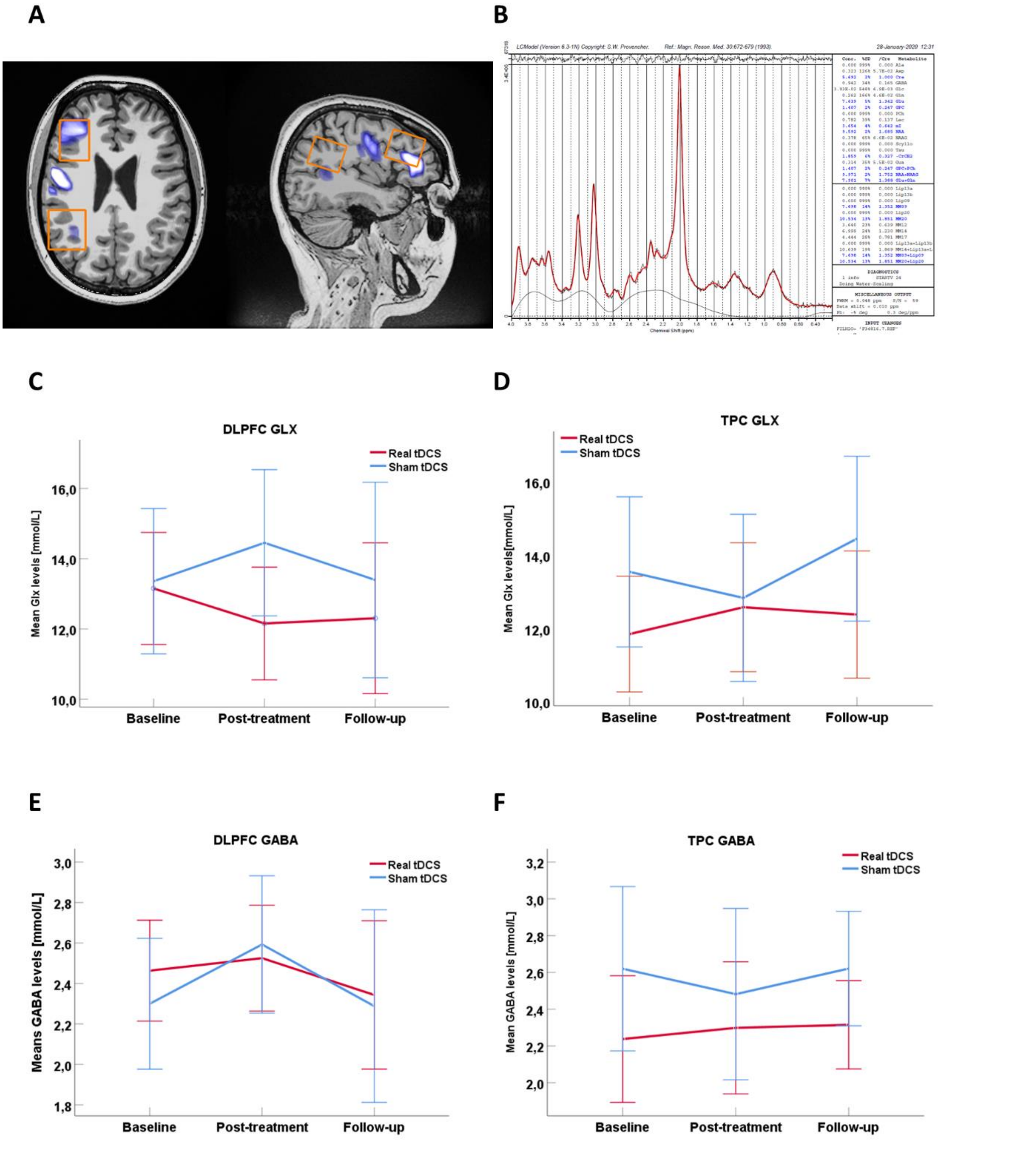

Notes. Panel A) Voxel placement (in orange) during MRS acquisition of the DLPC and TPC (sagittal and axial view) from one participant and the electrical field simulation for the group (in blue). Panel B) Typical successfully acquired MRS spectrum as given by LCModel. The black line denotes the measured data, the red line the model. Concentration estimates for the different neurotransmitters are given in the right-hand box. Mean Glx and GABA levels (institutional units of concentration, similar to mmol) in the DLPFC (panel C, E) and TPC (panel D, F) with AHRS as covariate. All Error bars denote 95% CI. Real tDCS in red, sham tDCS in blue.

Simultaneous Dichotic listening and fMRI paradigm

In the dichotic listening task, we found a significant main effect *Attentional focus* ($F_{(2,16)}$=5.38, *p*=.016, η_p^2=.402), with the highest accuracy rate in the no attentional focus condition (*M*=35.0 %, *SD*=1.1%), compared to attentional focus left (*M*=33.7 %, *SD*=1.5%) and right (*M*=34.6 %, *SD*=1.3%), and a significant interaction between *Attentional focus* and baseline AHRS scores ($F_{(2,16)}$=4.70, *p*=.025, η_p^2=.370).

Figure S5 below depicts a T-contrast across all three attentional focus conditions showing typical auditory cortex and temporo-parietal cortex activations during dichotic listening (p<.05 (FWE)).

Figure S5

T-contrasts for dichotic listening paradigm.

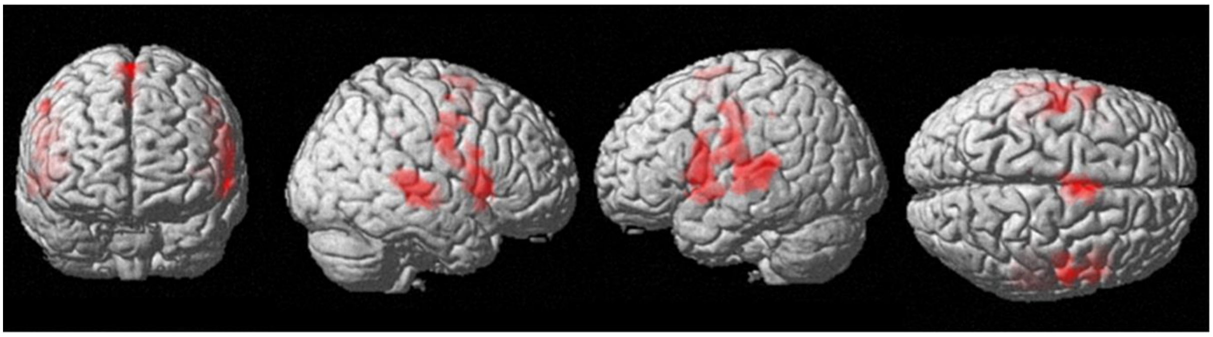

Simulation of electrical field

Figure S6

Electrical field simulation of tDCS for all participants in the real tDCS and sham tDCS groups.

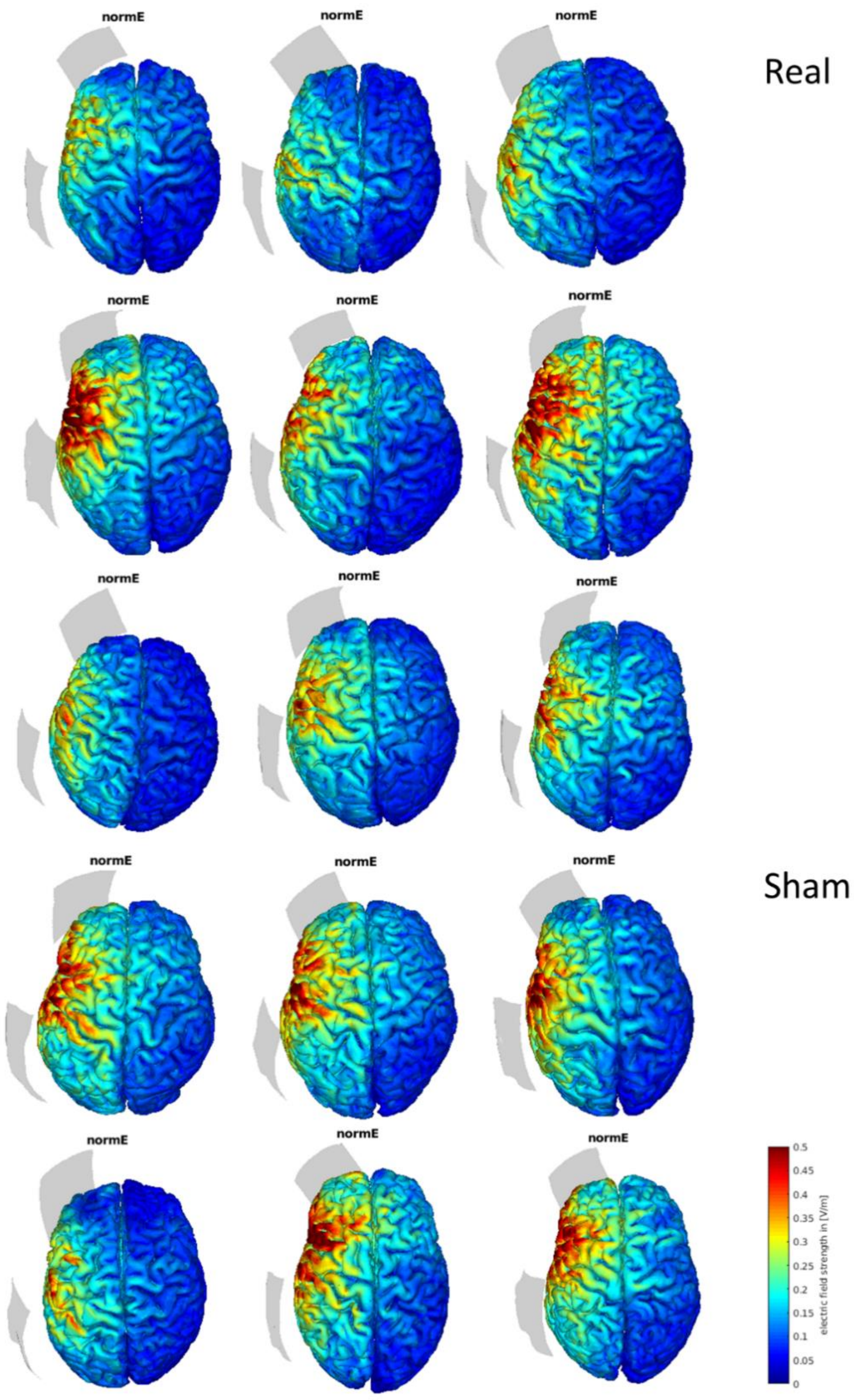

Notes. Electric fields (EF) (in V/m, maximum set on 0.5) for all participants receiving real and sham tDCS. Despite considerable inter-individual differences, no systematic differences between the two groups are observed (see main manuscript, section 4.6).

Correlations

In addition to the correlations provided in the main text, we explored whether changes in structural data were correlated with changes neurotransmitters. To this end, we computed correlations between structural changes at post-treatment and follow-up for all 12 chosen brain areas and changes in Glx and GABA at post-treatment and follow-up across all participants. None of these correlations were significant when corrected for multiple testing (all $rs \leq -.74$, $ps = .002$, $ps_{corr} = 1.0$).

To see if changes in structural data are associated with the simulated electric field, post-treatment and follow-up change in structural data for all 12 chosen brain areas were correlated with electric field and focality measures for those participants only who received real tDCS. None of these correlations were significant (all $rs \leq -.79$, $ps = .011$, $ps_{corr} = 1.0$) when corrected for multiple testing. Similarly, electric field and focality measures were correlated with Glx and GABA changes at post-treatment and follow-up in participants who received real tDCS. None were significant when corrected for multiple testing (all $rs \leq -.63$, $ps = .067$, $ps_{corr} = 1.0$). All corrections are based on the Holm's method [1].

Blinding, expectations, and side effects

In the real tDCS group, 8/11 participants (73%) and 4/10 participants (40%) in the sham group guessed correctly that they received real or sham tDCS (Fisher's Exact test, $p = .142$, one-sided). The number of correct guesses in the real tDCS group ($p = .227$) and sham group ($p = .754$) did not differ significantly from 50% as indicated by a binominal test. The experimenter guessed correctly for 5/11 (46%) of the participants in the real group and for 7/10 (70%) in the sham group (Fisher's Exact test, $p = .245$, one-sided). The number of total correct guesses did not differ significantly from 50% ($p = .664$).

Expectations for the treatment were generally high, with only 19% of participants expecting less than 5 on the 0-10 scale. Means in the real tDCS and sham group (M=6.8) did not differ significantly (see Table 1).

The number of participants reporting side effects are presented in Table 4 below. Chi-squared tests showed significant difference in neck pain (χ^2=25.37, *p*<.001), tingling sensation (all χ^2=22.60, *p*<.001) and red skin (all χ^2=29.10, *p*<.001) in reports of any of the side effects between real tDCS and sham groups, when mild, moderate, and severe reports were combined into "present" versus "absent". Note that neck pain was more reported in the sham group.

Participants did not guess significantly above chance level, but there was, descriptively, a tendency towards guessing that participants received real tDCS (*n*=13) versus guessing whether they received sham (*n*=8). This may be due to the tDCS-like sensations that participants experienced at the beginning of sham and, possibly, reflect participants' hope to receive real treatment. Nevertheless, it seems fair to conclude that the blinding worked reasonably well.

Table S3

Frequency of adverse events as measured with the tDCS Adverse Effects Questionnaire in number of occurrences in all real and sham tDCS sessions (n=210).

Adverse effects	Real tDCS (110 sessions)				Sham tDCS (100 sessions)				
	Mild	Moderate	Severe	∑	Mild	Moderate	Severe	∑	*p* for Chi^2
Headache	6	2	-	**8**	14	-	-	**14**	.031
Neckpain	1	-	-	**1**	9	10	2	**21**	**<.001**
Pain in Skull	9	5	-	**14**	10	-	-	**10**	.102
Tingling	40	22	-	**62**	21	3	1	**25**	**<.001**
Itching	31	7	-	**38**	19	2	-	**21**	.141
Burning sensation	35	3	-	**38**	18	7	2	**27**	.05
Red skin	34	1	-	**35**	2	-	-	**2**	**<.001**
Sleepiness	11	9	1	**21**	16	7	1	**24**	.474
Concentration	10	6	-	**16**	8	1	-	**9**	.242
Change in Humor	2	-	-	**2**	7	5	-	**12**	.005

CONSORT 2010 checklist of information to include when reporting a randomised trial*

Section/Topic	Item No	Checklist item	Reported on page No
**Title and abstract**			
	1a	Identification as a randomised trial in the title	1
	1b	Structured summary of trial design, methods, results, and conclusions	1
**Introduction**			
Background and objectives	2a	Scientific background and explanation of rationale	1-2
	2b	Specific objectives or hypotheses	2
**Methods**			
Trial design	3a	Description of trial design (such as parallel, factorial) including allocation ratio	6, table 2
	3b	Important changes to methods after trial commencement (such as eligibility criteria), with reasons	15
Participants	4a	Eligibility criteria for participants	3
	4b	Settings and locations where the data were collected	3
Interventions	5	The interventions for each group with sufficient details to allow replication, including how and when they were actually administered	3 and 5
Outcomes	6a	Completely defined pre-specified primary and secondary outcome measures, including how and when they were assessed	3
	6b	Any changes to trial outcomes after the trial commenced, with reasons	3
Sample size	7a	How sample size was determined	15
	7b	When applicable, explanation of any interim analyses and stopping guidelines	NA
Randomisation:			
	8a	Method used to generate the random allocation sequence	3-4

Sequence generation	8b	Type of randomisation; details of any restriction (such as blocking and block size)	3-4
Allocation concealment mechanism	9	Mechanism used to implement the random allocation sequence (such as sequentially numbered containers), describing any steps taken to conceal the sequence until interventions were assigned	3-4
Implementation	10	Who generated the random allocation sequence, who enrolled participants, and who assigned participants to interventions	3-4
Blinding	11a	If done, who was blinded after assignment to interventions (for example, participants, care providers, those assessing outcomes) and how	3-4
	11b	If relevant, description of the similarity of interventions	5
Statistical methods	12a	Statistical methods used to compare groups for primary and secondary outcomes	6-10
	12b	Methods for additional analyses, such as subgroup analyses and adjusted analyses	Supplementary Material (SM)
**Results**			
Participant flow (a diagram is strongly recommended)	13a	For each group, the numbers of participants who were randomly assigned, received intended treatment, and were analysed for the primary outcome	4, table 1
	13b	For each group, losses and exclusions after randomisation, together with reasons	4, table 1
Recruitment	14a	Dates defining the periods of recruitment and follow-up	6
	14b	Why the trial ended or was stopped	6
Baseline data	15	A table showing baseline demographic and clinical characteristics for each group	4, table 1
Numbers analysed	16	For each group, number of participants (denominator) included in each analysis and whether the analysis was by original assigned groups	5, figure 1
Outcomes and estimation	17a	For each primary and secondary outcome, results for each group, and the estimated effect size and its precision (such as 95% confidence interval)	10-14
	17b	For binary outcomes, presentation of both absolute and relative effect sizes is recommended	NA

Ancillary analyses	18	Results of any other analyses performed, including subgroup analyses and adjusted analyses, distinguishing pre-specified from exploratory	SM
Harms	19	All important harms or unintended effects in each group (for specific guidance see CONSORT for harms)	SM page 15-16
**Discussion**			
Limitations	20	Trial limitations, addressing sources of potential bias, imprecision, and, if relevant, multiplicity of analyses	15
Generalisability	21	Generalisability (external validity, applicability) of the trial findings	15
Interpretation	22	Interpretation consistent with results, balancing benefits and harms, and considering other relevant evidence	14-16
**Other information**			
Registration	23	Registration number and name of trial registry	3
Protocol	24	Where the full trial protocol can be accessed, if available	NA
Funding	25	Sources of funding and other support (such as supply of drugs), role of funders	16

* We strongly recommend reading this statement in conjunction with the CONSORT 2010 Explanation and Elaboration for important clarifications on all the items. If relevant, we also recommend reading CONSORT extensions for cluster randomised trials, non-inferiority and equivalence trials, non-pharmacological treatments, herbal interventions, and pragmatic trials. References to this checklist, see www.consort-statement.org.

References

1. Aickin, M. and H. Gensler, *Adjusting for multiple testing when reporting research results: the Bonferroni vs Holm methods.* American journal of public health, 1996. **86**(5): p. 726-728.
